# Evolutionary trajectory of undifferentiated connective tissue disease and impact of 2019 EULAR/ACR systemic lupus erythematosus classification criteria: insights from a longitudinal study

**DOI:** 10.1007/s10238-025-01668-1

**Published:** 2025-05-01

**Authors:** Claudia Ciancarella, Fulvia Ceccarelli, Licia Picciariello, Francesco Natalucci, Alessandra Ida Celia, Cristina Garufi, Silvia Mancuso, Giuseppe Tripodi, Simona Truglia, Angelica Gattamelata, Francesca Romana Spinelli, Cristiano Alessandri, Fabrizio Conti

**Affiliations:** Lupus Clinic, Rheumatology, Department of Clinical, Internal, Anesthesiologic and Cardiovascular, Sciences, Sapienza University of Rome, Viale del Policlinico 155, 00161 Rome, Italy

**Keywords:** Undifferentiated connective tissue disease, Systemic lupus erythematosus, Classification criteria, Evolution

## Abstract

Undifferentiated connective tissue disease (UCTD) is a condition characterized by serological evidence of autoimmunity and occurrence of clinical symptoms suggestive for systemic autoimmune diseases, yet not fulfilling specific classification/diagnostic criteria. In the present longitudinal, observational, retrospective study, we aimed at analysing the evolution of UCTD course, focussing on the impact of 2019 EULAR/ACR classification criteria for systemic lupus erythematosus (SLE). Since 2008 we consecutively collected data about UCTD patients. All subjects were evaluated every six months, to record the development of clinical and laboratory features suggestive for specific autoimmune diseases. Finally, we retrospectively applied the 2019 EULAR/ACR SLE classification criteria at the first and last visit in our outpatient clinic. All the patients included in the study had been evaluated at our Lupus Clinic before the release of 2019 EULAR/ACR criteria. We included 201 UCTD patients [F/M 191/10, median age at first visit 46 years (IQR 21), median disease duration at first visit 3 years (IQR 9)]. At the first visit, 27 patients (13.4%) already met 2019 EULAR/ACR SLE classification criteria. Logistic regression analysis demonstrated the association between SLE classification and thrombocytopenia, anti-dsDNA/anti-Sm positivity, low C4 levels, joint involvement. During a mean observation period of 45.9 ± 35.6 months, 18.9% of patients were lost to follow-up, while 141 patients were followed. At last visit, additional 11 patients (7.8%) could be classified as having SLE. A relevant proportion of UCTD patients could be reclassified as having SLE according to the most recent classification criteria. Thrombocytopenia, anti-DNA/anti-Sm positivity and low C4 levels represent the most associated factors.

**Supplementary Information:**

The online version contains supplementary material available at 10.1007/s10238-025-01668-1.

## Introduction

Undifferentiated connective tissue disease (UCTD) has been defined as a condition characterized by serological evidence of autoimmunity and occurrence of clinical symptoms suggestive for systemic autoimmune diseases, but not fulfilling specific criteria for a connective tissue disease (CTD) [1].

The term UCTD was first introduced in 1978 to indicate the coexistence of typical manifestations of the classical rheumatological diseases [2]. Few years later, Leroy introduced the term *'undifferentiated connective tissue syndromes'*, referring to CTD in their early stages, underlining the possible lack of specificity in disease manifestations in this phase [3]. However, longitudinal evaluation of UCTD patients clearly demonstrated as not all cases represented early stages of definite CTD. Indeed, today we know that under the umbrella term of UCTD there are several serological and clinical conditions with different possible evolutions: some cases progress into a defined autoimmune disease, while other cases remain stable or even undergo a complete resolution [4].

In this field, a literature review conducted by Mosca and colleagues in 2023 demonstrated as only 30% of UCTD patients will develop a definite CTD during follow-up and most frequently within the first 5 years from the disease onset [5]. According to published data, the evolution usually occurs to systemic lupus erythematosus (SLE) but also to other autoimmune diseases, prevalently systemic sclerosis (SSc) and Sjogren's syndrome (SS) [5]. On the other hand, 70% of patients do not progress and can be defined as having 'stable UCTD' that mainly affect the female sex in the 3rd and 4th decade of life, and it is generally characterized by a simplified autoantibody profile and by a mild clinical picture, without major organ involvement.

Specific criteria for UCTD were proposed in 1999 and included: (i) signs and symptoms suggestive of CTD, but not fulfilling the criteria for defined CTD, (ii) positivity for antinuclear antibodies and (ii) disease duration of at least 3 years [4].

However, lack of consensus on diagnostic/classification criteria has prevented obtaining more specific data on epidemiological and demographic features of UCTD. As the evolution to a defined CTD can occur, it is important to follow these patients regularly, especially during the first years from disease onset. Furthermore, it is relevant to identify the features that can predict the evolution. Data from the literature have suggested several candidate factors. For instance, some factors have been associated with evolution into SLE: among these, fever, discoid lupus, alopecia, serositis, photosensitivity, positivity for anti-dsDNA/anti-Sm and anticardiolipin, and the presence of multiple specific autoantibodies [6].

The interest in UCTD stems from the evidence that this condition is frequently encountered in clinical practice. Indeed, 20–50% of patients presenting to a rheumatology department are diagnosed with UCTD [7, 8].

In last decades, the introduction of new classification criteria for defined CTDs, focussing on their early detection, has undoubtedly had an impact on the incidence of UCTD. This is the case of 2019 EULAR/ACR classification criteria for SLE [9] that combine higher sensitivity and specificity values than the previous criteria [10, 11]*.* Therefore, some patients, who would previously have been diagnosed with UCTD, may have a diagnosis of a specific CTD, even in the early stages of the disease.

The distinction between diagnosis and classification represents a crucial point in several diseases and is still being widely discussed. Indeed, classification criteria arise from the need in creating homogeneous cohorts of patients that can be enrolled in clinical studies and randomized controlled trials. Conversely, according to Fries-Holman's diagnostic principle, the diagnosis allows to identify patients for the purpose of appropriately treating patients [12]. The introduction of new classification criteria is generally followed by studies designed to evaluate their sensitivity and specificity in earlier cohorts. This was already the case for the 2012 SLICC criteria, as reported in the studies conducted in Swedish cohorts [11, 13, 14].

Moving from these premises, we conducted a longitudinal, observational, retrospective study in order to analyse the evolution of UCTD, with special emphasis on the application of 2019 EULAR/ACR classification criteria for SLE.

### Patients and methods

Since 2008 we have collected data about patients diagnosed with UCTD, attending at the Lupus Clinic of the Rheumatology Unit, Sapienza University of Rome.

Here, we performed a retrospective analysis of prospectively collected data from a large cohort of UCTD patients. For this purpose, clinical, laboratory and therapeutical data were collected in a standardized computerized electronically filled form, including demographics, past medical history, date of diagnosis, comorbidities, previous and concomitant treatments, serological status [C3/C4 levels (radial immunodiffusion), ANA (IIF on HEp-2), anti-dsDNA (IIF on Crithidia Luciliae), anti-Ro/SSA, anti-La/SSB, anti-Sm, and anti-RNP, anticardiolipin (anti-CL, IgG/IgM) and anti-β2 Glycoprotein-I (anti-β2GPI IgG/IgM) (ELISA assay), lupus anticoagulant (LA) (according to the guidelines of the International Society on Thrombosis and Haemostasis)]. All subjects were evaluated every six months or in accordance with the patient’s clinical course, to record the development of clinical and laboratory features suggestive for specific autoimmune diseases.

The present analysis was restricted to UCTD patients with a follow-up in our clinic of at least 6 months.

We retrospectively applied the 2019 EULAR/ACR classification criteria for SLE, at the first and last visit in our outpatient clinic. In addition, we analysed the clinical evolution of UCTD patients towards other definite CTDs during our observation. All the patients included in the study had been evaluated at our Lupus Clinic before the release of 2019 EULAR/ACR criteria.

#### Statistical analysis

The statistical analyses were performed using version 5.0 of the GraphPad statistical package. Normally distributed variables were summarized using the mean ± standard deviation (SD), and non-normally distributed variables by the median and interquartile range (IQR). Frequencies were expressed by percentage. Wilcoxon’s matched pairs test and the paired t-test were performed accordingly. Univariate comparisons between nominal variables were calculated using the chi-square test or Fisher’s exact test, where appropriate. Spearman’s test was used to test correlation. Furthermore, multivariate linear regression analysis was performed (Statistical Package for Social Sciences—SPSS 20, Chicago, IL, USA). Two- tailed p-values were reported; p-values less than 0.05 were considered significant.

## Results

Our analysis included 201 patients diagnosed with UCTD at the first visit in our outpatient clinic. They were mainly female (F/M 191/10) with a median age at first visit of 46 years (IQR 21), median disease duration at first visit of 3 years (IQR 9; mean ± SD 6.5 ± 7.5 years)].

At first evaluation in our outpatient clinic, the most frequent manifestation was joint involvement, with the presence of inflammatory arthralgia/arthritis in 145 patients (72.1%), followed by skin and haematological manifestations (37.8% and 19.4%, respectively). Moving on laboratory features, the most frequent immune/autoimmune abnormalities were the positivity for antiphospholipid antibodies, observed in 50 patients (24.9%), followed by reduction in C3 serum levels (10.4%), 12 patients (5.9%) showed positivity for anti-dsDNA and/or anti-Sm antibodies.

Focussing on treatment, 118 patients (58.7%) were treated by hydroxychloroquine (HCQ), 36 (17.9%) by glucocorticoids (GCs). Only six patients (2.9%) were taking immunosuppressant drugs at the first visit.

**Fig. 1:**
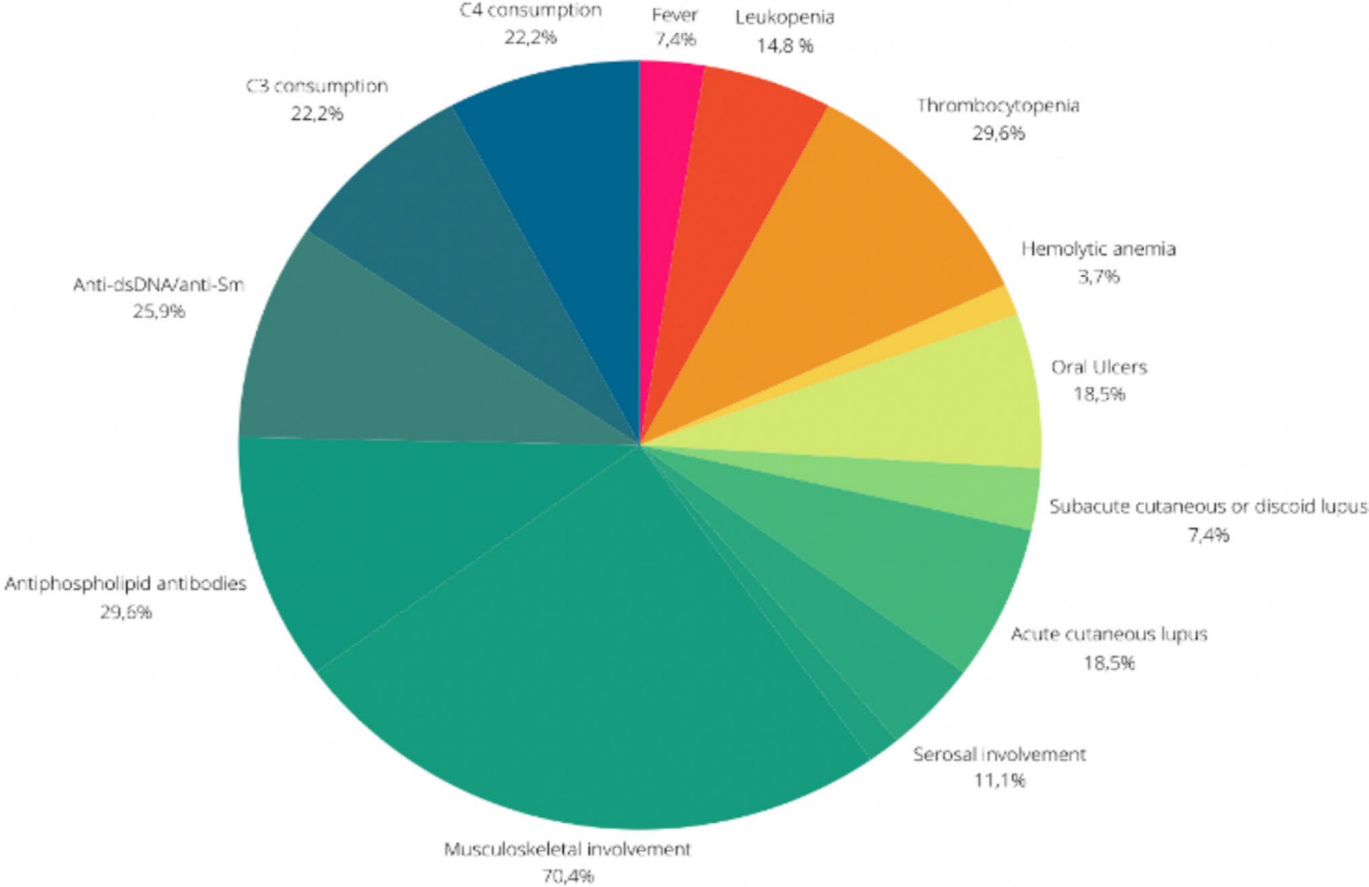
Clinical and laboratory manifestations of SLE patients (*N* = 27) reclassified at first visit according to 2019 EULAR/ACR criteria

By retrospectively applying the 2019 SLE EULAR/ACR classification criteria at the first visit, 27 patients (13.4%) could be classified as affected by SLE. In these reclassified patients, the most frequent clinical manifestation was joint involvement (70.4%), followed by thrombocytopenia (29.6%) and positivity for antiphospholipid antibodies (29.6%) (Fig. 1). In these patients, the prevalence of anti-dsDNA/anti-Sm positivity was equal to 25.9%. Immunological and clinical domains of these patients, according to classification criteria, are detailed in Table S1.

We found a significant association between SLE reclassification and thrombocytopenia (*p = *0.0001), serositis (*p = *0.02), joint involvement (*p = *0.03), low levels of C3 and C4 (*p = *0.03, *p = *0.0001, respectively), positivity for anti-dsDNA/anti-Sm (*p = * < 0.0001). Logistic regression analysis confirmed the association with thrombocytopenia (*p < *0.0001, Exp(B) 0.39, SE 0.75, 95%CI 0.09–0.171), anti-dsDNA/anti-Sm positivity (*p = *0.01, Exp(B) 0.05, SE 0.91, 95%CI 0.01–0.326), low C4 levels (*p = *0.001, Exp(B) 0.05, SE 0.91, 95%CI 0.01–0.326), joint involvement (*p = *0.01, Exp(B) 0.227, SE 0.58, 95%CI 0.07–0.71). At the first visit, among the 27 SLE reclassified subjects, 16 patients (59.2%) were taking a treatment (HCQ 12 patients, GCs 4 patients). During longitudinal observation, 5 patients (18.5%) were lost to follow-up. The remaining 22 patients were followed up for a median period of 43.5 months (IQR 37.7). Compared with the first visit, the proportion of treated patients increased to 95.4%, with the introduction of immunosuppressive agents in addition to HCQ in 4 patients due to other disease-related manifestations occurred during the follow-up. In detail, one patient was treated by Cyclosporin A (CyA) for skin manifestations, one patient by CyA and belimumab (BLM) due to refractory skin and joint involvement, one with rituximab (RTX) due to severe thrombocytopenia, one with mycophenolate mofetil (MMF) and RTX due to the presence of refractory renal involvement (class IV lupus nephritis). Furthermore, we observed a quite increase in the proportion of patients treated by GCs when comparing first and last visit (14.8% and 22.7%, respectively).

We also evaluated the presence of damage. Indeed, according to the SLICC damage index (SDI) [15], at the first visit 2 patients (7.4%) showed damage in at least one organ/system; this proportion increased to 18.2% at the last assessment.

**Fig. 2:**
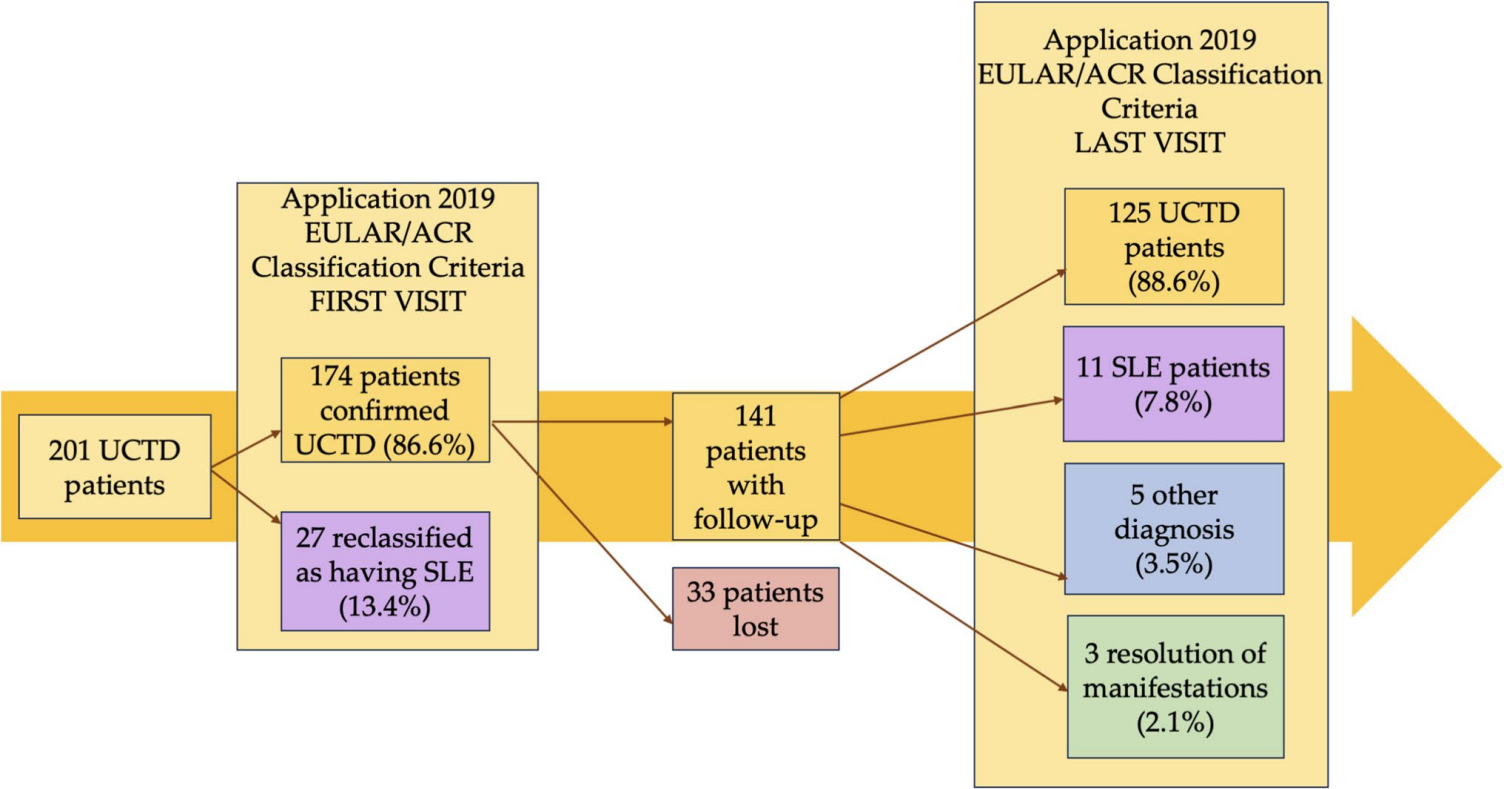
Follow-up of UCTD patients (*N* = 201)

The remaining 174 UCTD patients were longitudinally followed: the evolutionary trajectory is graphically presented in Fig. 2. In detail, during a mean follow-up of 45.9 ± 35.6 months, 33 patients (18.9%) were lost to follow-up, while 141 patients continued to attend regular outpatient clinics.

The application of 2019 SLE EULAR/ACR classification criteria at last available visit for these 141 patients allowed to identify additional 11 subjects (7.8%) that could be classified as having SLE due to the appearance of new clinical/laboratory features. Immunological and clinical domains of these patients, according to classification criteria, are detailed in Table S2. When compared with stable UCTD, SLE reclassified patients showed significant higher prevalence of joint involvement (81.8% versus 54.6%, *p = *0.0001), thrombocytopenia (18.2% versus 6.9%, *p = *0.03), positivity for anti-dsDNA (36.4% versus 2.3%, *p = *0.0001) and anti-Sm (18.2% versus 1.3%, *P = *0.001), low C3 levels (36.4% versus 3.8%, *p = *0.001). Moving on treatment, 10 patients (90.0%) were taking HCQ, 3 patients (27.3%) were taking GCs and only one patient was treated by methotrexate for joint involvement.

Furthermore, other patients were reclassified: three as having SS, two as having psoriatic arthritis and one as affected by rheumatoid arthritis (according to specific classification criteria) [16–18].

Considering the remaining patients, 125 subjects (88.6%) showed a stable condition of UCTD and 3 patients (2.1%) showed a complete resolution of clinical and serological manifestations at last visit in our outpatient clinic.

## Discussion

In the present longitudinal, observational, retrospective study, we aimed at applying the 2019 EULAR/ACR classification criteria for SLE in a large UCTD cohort. Overall, about 20% of patients could be reclassified as having SLE at first visit in our outpatient clinic or during the observational period, due to the appearance of additional serological and/or clinical features. Furthermore, during the follow-up, it was possible to classify additional 5 patients as affected by other rheumatological conditions. Finally, a small proportion of patients (≅ 2% of subjects) experienced a complete and persistent remission of clinical and serological features. Then, in our cohort, a condition of stable UCTD was observed in about 80% of patients after an observation lasting 4 years.

The condition of UCTD has traditionally been considered a vague concept, including all conditions that do not meet the criteria for a specific disease. Nonetheless, UCTD could also reflect the early phase of CTD or a transitory condition that could completely resolve. The growing availability of longitudinal studies certainly helped to better elucidate the evolution of UCTD. Furthermore, the introduction of increasingly sensitive classification criteria for CTDs has made it possible to reduce the proportion of early-stage CTDs that fall under the UCTD umbrella, making possible to earlier classify patients.

It has been clearly demonstrated as the early identification of CTDs is essential for more accurate therapeutic approach and for improving patient’s prognosis, since there are no clinical practice guidelines or recommendations for UCTD [19].

Our analysis showed that a significant proportion of patients previously classified as having UCTD could already be classified as having SLE at the first visit according to the 2019 EULAR/ACR criteria. These results agree with previous data from the literature, that overall report reclassification to a definite CTD, especially SLE, in about 30% of patients after an observation period ranging from 3 to 7 years [4, 20]. In particular, the rates of evolution towards definite CTDs observed in our cohort are in line with other studies conducted on Italian population [21, 22].

Furthermore, we attempted to identify some clinical and laboratory features associated with reclassification. Overall, joint involvement and thrombocytopenia from a clinical point of view, positivity for anti-dsDNA/anti-Sm antibodies and low C3/C4 serum levels from a serological point of view, were significantly more frequent in reclassified patients at first or last evaluation. These factors are the same described in the literature, although it should be considered that the criteria considered for reclassification are different, depending on the period in which the studies were conducted [23]. Undoubtedly, the identification of features associated with evolution towards a definite disease is very important because it might allow to stratify the risk of progression and thus to establish tighter controls. A greater knowledge of this aspect will certainly come from specifically designed prospective studies [24].

Furthermore, the possibility of early identification of CTD is relevant because the term *'undifferentiated'* could create a sense of bewilderment in patients, which is often associated with less trust in the clinician. Patients may also tend to downplay the importance of the condition, with consequent reduction in treatment adherence and in follow-up. Indeed, in our court 16.4% of the patients stopped making regular outpatient visits.

Our analysis also provides data about the treatment of patients with UCTD. A relevant proportion of patients received a treatment at first visit in our outpatient clinic, represented in most cases by antimalarial drugs; less than 20% of patients received GCs and only few subjects were taking immunosuppressant drugs.

The therapeutical scenario completely changes when we considered the SLE reclassified patients. Indeed, at last available visit, almost all the patients were taking a treatment, and, in some cases, immunosuppressive or biological drugs were introduced due to the presence of more severe manifestations of disease. Furthermore, the application of SDI demonstrated the accrual of chronic damage during the follow-up, with a significant increase of patients with damage in at least one organ/domain after 4-year follow-up. This appears to be extremely interesting, as although these patients have a mild serologic and clinical profile, they can nevertheless develop damage and sometimes require aggressive treatment. This therefore underlines the need for close monitoring. Obviously, this damage index has only been applied in SLE reclassified patients as it has not been validated for UCTD.

Certainly, our study shows some limitations. First of all, the referral to a Lupus Clinic could influence the patient phenotype, because it is likely to be mainly subjects with lupus-like features. Secondly, retrospective studies are always marred by the absence of complete clinical information of all patients.

In conclusion, our data confirm that UCTD represents an evolving condition, with the possible evolution to definite forms of CTDs, sometimes with severe pictures requiring second-line therapy. However, complete resolution of the condition can be observed, albeit in a small percentage of patients. Certainly, we are waiting for studies with larger cohort and with homogeneity of the applied classification criteria. 

## Supplementary Information

Below is the link to the electronic supplementary material.

Supplementary file1 (DOCX 22 KB)

## Data Availability

Data are provided within the manuscript.
