# Supplementary material for: Evolutionary trajectory of undifferentiated connective tissue disease and impact of 2019 EULAR/ACR systemic lupus erythematosus classification criteria: insights from a longitudinal study
Source: Clin Exp Med. 2025 May 1;25(1):134. doi: 10.1007/s10238-025-01668-1 (PMC12045818; doi:10.1007/s10238-025-01668-1)
Supplement: Supplementary file 1 — Supplementary file1 (DOCX 22 KB) [file 10238_2025_1668_MOESM1_ESM.docx]

**Table S1: Patients reclassified as having SLE at the first visit.**

| **Patients** | **Immunological Domains** | **Clinical domains** |
| --- | --- | --- |
| **1** | ANA, aPL | Joint involvement  (tenderness in 2 or more joints and at least 30 minutes of morning stiffness)  Thrombocytopenia |
| **2** | ANA | Joint involvement  (synovitis involving 2 or more joints characterized by swelling or effusion)  Pleural or pericardial effusion |
| **3** | ANA, aPL, anti-Sm,  low C4 levels | Joint involvement  (synovitis involving 2 or more joints characterized by swelling or effusion)  Fever |
| **4** | ANA, aPL, low C3 levels | Joint involvement  (synovitis involving 2 or more joints characterized by swelling or effusion) |
| **5** | ANA,  low C3 and C4 levels | Joint involvement  (synovitis involving 2 or more joints characterized by swelling or effusion)  Fever |
| **6** | ANA | Joint involvement  (synovitis involving 2 or more joints characterized by swelling or effusion)  Pleural or pericardial effusion |
| **7** | ANA, aPL | Joint involvement  (tenderness in 2 or more joints and at least 30 minutes of morning stiffness) |
| **8** | ANA | Thrombocytopenia  Acute cutaneous lupus |
| **9** | ANA | Joint involvement  (tenderness in 2 or more joints and at least 30 minutes of morning stiffness)  Acute cutaneous lupus |
| **10** | ANA, aPL | Joint involvement  (synovitis involving 2 or more joints characterized by swelling or effusion)  Oral ulcers  Autoimmune hemolysis |
| **11** | ANA | Joint involvement  (synovitis involving 2 or more joints characterized by swelling or effusion)  Acute cutaneous lupus |
| **12** | ANA,  low C3 and C4 levels | Joint involvement  (synovitis involving 2 or more joints characterized by swelling or effusion) |
| **13** | ANA | Joint involvement  (synovitis involving 2 or more joints characterized by swelling or effusion)  Oral ulcers  Leukopenia |
| **14** | ANA | Joint involvement  (tenderness in 2 or more joints and at least 30 minutes of morning stiffness)  Pleural or pericardial effusion |
| **15** | ANA | Joint involvement  (tenderness in 2 or more joints and at least 30 minutes of morning stiffness)  Thrombocytopenia  Non-scarring alopecia |
| **16** | ANA, antidsDNA, aPL  low C3 and C4 levels | Joint involvement  (synovitis involving 2 or more joints characterized by swelling or effusion) |
| **17** | ANA | Joint involvement  (synovitis involving 2 or more joints characterized by swelling or effusion)  Thrombocytopenia |
| **18** | ANA, antidsDNA | Thrombocytopenia |
| **19** | ANA, antidsDNA | Thrombocytopenia |
| **20** | ANA, antidsDNA | Joint involvement  (tenderness in 2 or more joints and at least 30 minutes of morning stiffness)  Acute cutaneous lupus |
| **21** | ANA, antidsDNA, aPL  low C4 levels | Pleural or pericardial effusion  Joint involvement  (tenderness in 2 or more joints and at least 30 minutes of morning stiffness)  Proteinuria |
| **22** | ANA,  low C3 levels | Joint involvement  (synovitis involving 2 or more joints characterized by swelling or effusion)  Thrombocytopenia |
| **23** | ANA | Thrombocytopenia, Leukopenia  Acute cutaneous lupus |
| **24** | ANA | Joint involvement  (synovitis involving 2 or more joints characterized by swelling or effusion)  Oral ulcers  Pleural or pericardial effusion |
| **25** | ANA,  Low C3 levels | Leukopenia  Acute cutaneous lupus |
| **26** | ANA | Joint involvement  (synovitis involving 2 or more joints characterized by swelling or effusion)  Acute cutaneous lupus |
| **27** | ANA  Low C3 and C4 levels | Joint involvement  (synovitis involving 2 or more joints characterized by swelling or effusion) |

**Legend** - ANA: antinuclear antibodies; aPL: antiphospholipid antibodies

**Table S2: Patients reclassified as having SLE during the follow-up**

| **Patients** | **Immunological Domains** | **Clinical domains** |
| --- | --- | --- |
| **1** | ANA | Joint involvement  (synovitis involving 2 or more joints characterized by swelling or effusion)  Oral ulcers |
| **2** | ANA, anti-Sm | Joint involvement  (synovitis involving 2 or more joints characterized by swelling or effusion)  Oral ulcers  Acute cutaneous lupus |
| **3** | ANA, antidsDNA | Oral ulcers  Joint involvement  (tenderness in 2 or more joints and at least 30 minutes of morning stiffness) |
| **4** | ANA | Joint involvement  (synovitis involving 2 or more joints characterized by swelling or effusion)  Thrombocytopenia |
| **5** | ANA, aPL  Low C3 levels | Joint involvement  (tenderness in 2 or more joints and at least 30 minutes of morning stiffness) |
| **6** | ANA, aPL | Joint involvement  (tenderness in 2 or more joints and at least 30 minutes of morning stiffness)  Leukopenia |
| **7** | ANA, aPL  Low C4 levels | Joint involvement  (synovitis involving 2 or more joints characterized by swelling or effusion)  Thrombocytopenia |
| **8** | ANA, aPL, antidsDNA | Joint involvement  (tenderness in 2 or more joints and at least 30 minutes of morning stiffness) |
| **9** | ANA, aPL  Low C3/C4 levels | Joint involvement  (tenderness in 2 or more joints and at least 30 minutes of morning stiffness) |
| **10** | ANA | Fever  Oral ulcers  Pleural or pericardial effusion |
| **11** | ANA, antiSm  Low C3 levels | Pleural or pericardial effusion |

**Legend** - ANA: antinuclear antibodies; aPL: antiphospholipid antibodies
